# Acute Cholecystitis from Biliary Lithiasis: Diagnosis, Management and Treatment

**DOI:** 10.3390/antibiotics12030482

**Published:** 2023-02-28

**Authors:** Maria Ludovica Costanzo, Vito D’Andrea, Augusto Lauro, Maria Irene Bellini

**Affiliations:** Department of Surgery, Sapienza University of Rome, Viale Regina Elena 324, 00161 Rome, Italy

**Keywords:** acute cholecystitis, antibiotics, cholecystectomy, biliary lithiasis

## Abstract

Biliary lithiasis is a global disorder affecting nearly 20% of the world’s population, although most cases occur without symptoms. Gallbladder stones could move into the common bile duct after gallbladder contraction, causing acute cholecystitis. The progression of the acute disease can take different forms, from mild inflammation, treatable with oral antibiotics, to the most severe forms with septic shock or biliary peritonitis, requiring specific treatment. Liver function tests and abdominal ultrasound are generally sufficient for diagnostic purposes. The most commonly used antibiotic is penicillin, with piperacillin achieving the best results; alternatively, fluoroquinolones could also be used, although there is no univocal consensus and surgery remains the only definitive treatment. A prolonged antibiotic therapy after cholecystectomy seems inadvisable, except in severe cases and/or in the immuno-compromised patient, where it should be periodically evaluated to avoid antibiotic resistance and unnecessary use. This review presents an evidence-based analysis to describe the advantages and disadvantages of the available options for the treatment of biliary lithiasis and cholecystitis, from the pathophysiological mechanisms behind lithiasis formation and also covering the main diagnostic findings for biliary stones, recommending an approach tailored to the patient’s characteristics and to the team’s expertise.

## 1. Introduction

Biliary lithiasis is the most common disease in the world, affecting the gallbladder and biliary tree, in about 20% of the general population, although only few show symptoms clinically [1]. According to a systematic review by Wang et al. [2], more than 25 million people in the United States suffer from cholelithiasis, with healthcare expenditures of USD ten billion annually [3]. Additionally, as many as 15% of patients with cholelithiasis are also affected by concomitant choledocholithiasis [4]. More specifically, in developed countries, about 10% of adults and 20% of people > 65 years old have gallstones; however, in Italy, in particular, the incidence of choledocholithiasis in patients with gallbladder stones is 5–10%, with a 4–5% incidence of previously undetected choledocholithiasis, when routine choledochography is performed [5,6,7]. There are other data in the more recent literature [8,9] that state choledocholithiasis has been detected in about 4–18% of patients undergoing cholecystectomy (CCY) [10,11]. It is estimated that about 15% of the American population suffers from gallstones, with more than 700,000 CCYs performed each year; 10–15% of these cases have concomitant common bile duct stones (CBDS) [12]. In Japan, the overall incidence of gallstones is about 10% [13].

The prevalence of choledocholithiasis in patients with cholelithiasis increases with age: for those younger than 60 years, it is 8–15%, while it rises to 15–60% for those older than 60 years [14,15,16]. Cholelithiasis is more common in women, especially during pregnancy, elderly patients, and those with dyslipidemia. Cholesterol stones are a common trigger in obese patients with sedentary lifestyle habits or in patients who have recently and intentionally lost weight.

Acute cholecystitis (ACC) is caused by an obstruction of the cystic duct, eventually determining biliary stasis, which, in turn, initiates a cascade of inflammatory changes, affecting the gallbladder epithelium to the point where it becomes necrotic, and thus could turn into a full-blown infection. Given this overgrowth, the puncture of the necrotized wall for drainage purposes could result in an invasion of enteric organisms and bacteria [17,18,19], among which the commonest are Escherichia coli, Klebsiella, Enterococcus, and anaerobic germs [20]. It has been considered that in patients undergoing cholecystectomy for ACC, the presence of bacteria in the bile varies between 41% and 63% [21,22,23,24,25]. Jarvinen et al. [26] observed in their study of 515 patients with ACC that bile culture was positive in 63% of cases after 24 h of symptoms, while after 11 days of evolution, this percentage dropped to 31%.

Most CBDS are formed in the gallbladder and move into the common bile duct (CBD) after gallbladder contraction. Although stones can reach the duodenum, following the bile flow, due to the smaller diameter of the CBD distal to Vater’s papilla, these often are trapped, obstructing the main bile duct—a risk factor for ascending cholangitis.

The obstruction of the bile and pancreatic ducts are presumed to increase intraductal pressure, potentially triggering pancreatic enzyme activation, resulting in acute biliary pancreatitis [27,28].

## 2. Pathophysiology

**Figure 1:**
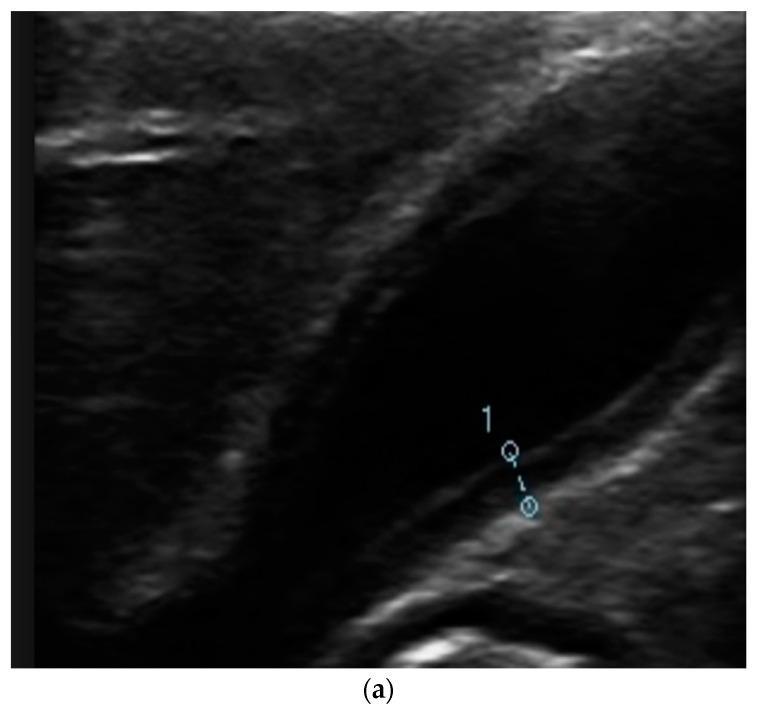

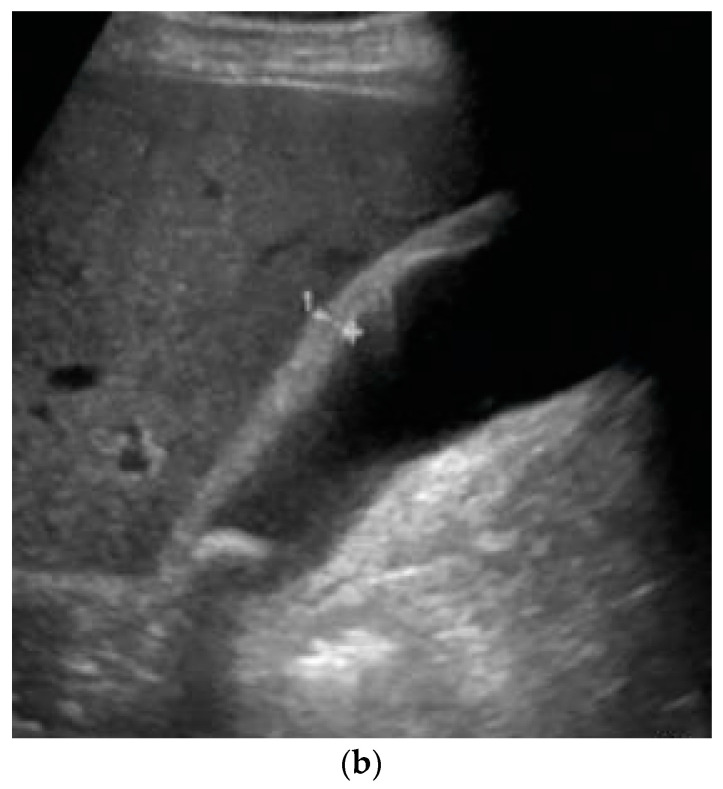
(**a**,**b**). Thickened-wall gallbladder with “Rail-like” feature, containing minimal biliary sludge mixed with a 7 mm calcinotonic formation at the infundibulum.

Biliary sludge, which is composed mainly of a polymer of bilirubin, calcium bilirubinate, cholesterol microcrystals, and mucin, is, in the majority of cases, the precursor of gallstones (Figure 1). Its formation begins during cholecystic stasis, occurring both in physiological and pathological situations, as, for example, pregnancy or total parenteral nutrition. In most cases it is asymptomatic and disappears when the primary condition resolves. Alternatively, biliary sludge can evolve into gallstones or migrate into the biliary tree, obstructing ducts and leading to biliary colic, cholangitis, or pancreatitis.

There are several types of gallstones [29]: cholesterol stones account for more than 85% of cholecystic stones in Western countries. These types of stones tend to form when the bile is oversaturated with cholesterol. Normally, water-insoluble cholesterol is made water-soluble with the combination of bile salts and lecithin, forming mixed micelles. When large amounts of cholesterol [30] are absorbed by the supersaturated bile excreted from the epithelial cells in the gallbladder, this then translates into an impairment of the gallbladder emptying and stones formation. The excess of cholesterol is then converted to cholesterol esters and stored in the mucosa and lamina propria, stiffening the sarcolemmal membrane of the smooth muscle cells. At that point, the resulting chronic inflammation of the wall means fibrosis, so practically the organ contraction is lost. In other words, the longer cholesterol supersaturated bile is in the lumen of the gallbladder, the more there is a risk that it will crystallize and thus give first a microlithiasis and, later on, macrolithiasis.

Oversaturation of bile with cholesterol is often related to excessive cholesterol secretion, as it occurs in conditions such as diabetes or obesity. This can also be caused by a reduced secretion of bile salts, such as in cystic fibrosis, due to the malabsorption of bile salts, or when there is reduced lecithin secretion, as happens in a genetic disorder causing a form of progressive familial intrahepatic cholestasis.

Excess cholesterol precipitates in the solution as solid microcrystals, and it is accelerated by mucin, a glycoprotein, or other proteins in the bile. The microcrystals then aggregate and increase in volume. This is a process facilitated by the binding capacity of mucin to form scaffolds and by the retention of the microcrystals in the gallbladder, whose contractility is impaired because of the excess of cholesterol in the bile.

In contrast, black pigment stones are small and hard, formed by calcium bilirubinate and inorganic calcium salts, such as calcium carbonate and calcium phosphate. Factors facilitating stone development include alcoholic hepatopathy, chronic hemolysis, and advanced age.

Pigmentary brown stones are soft, greasy, and are mainly composed of bilirubinate and fatty acids, such as calcium palmitate or calcium stearate [15,16]. They form during infection, inflammation, and parasitic infestation, including that of liver flukes in Asia.

Gallstones grow about 1–2 mm/year, taking 5 to 20 years to become large enough to cause problems. Most stones form in the gallbladder, but brown ones form in the biliary tract. The stones may migrate into the main biliary pathway after cholecystectomy or, especially in the case of pigmentary brown stones, may form upstream of a stenosis, due to biliary stasis.

## 3. Symptoms

Biliary colic and cholecystitis have a very similar symptomatology [31,32], in relation to the quality and location of pain, although ACC is usually described with pain of longer duration (>6 h) and greater severity. Vomiting is frequent, as it is the right hypochondrium tenderness, within a few hours of the positivity of Murphy’s sign (deep inspiration exacerbating pain on palpation of the cystic point at the right hypochondrium and blocking inspiration), along with an involuntary defense reaction of the right upper quadrant abdominal muscles. Usually, mild fever is common. Right upper quadrant pain with nausea and vomiting in patients with choledocholithiasis is probably due to extrahepatic bile duct distension [28] rather than gallbladder distension due to cystic duct obstruction. In the elderly, the first or only symptoms may be systemic and non-specific, including poor appetite, vomiting, malaise, and weakness. Without treatment, 10% of patients develop localized perforation, and 1% free perforation with peritonitis. Severe abdominal pain, high fever and intense chills, rigidity with Blumberg’s sign positivity, or paralytic ileus are indicative of empyema (pus) in the gallbladder, gangrene, or perforation. If ACC is associated with jaundice or cholestasis, most likely a partial obstruction of the main biliary tract, usually due to stones or inflammation, occurred.

It is deemed that up to 25% of initially asymptomatic individuals will develop biliary colic within 10 years [33]. The onset of pain heralds the onset of recurrent symptoms in most patients, allowing an early identification of those at risk of developing major complications, such as pancreatitis, cholecystitis, and biliary obstruction.

### Complications

In patients with initially silent gallstones, complications may occur in 2–3% of patients within 10 years [34]. About 10–33% of patients with symptomatic cholelithiasis present with CBDS, depending on the age of the patient [10,35].

**Figure 2:**
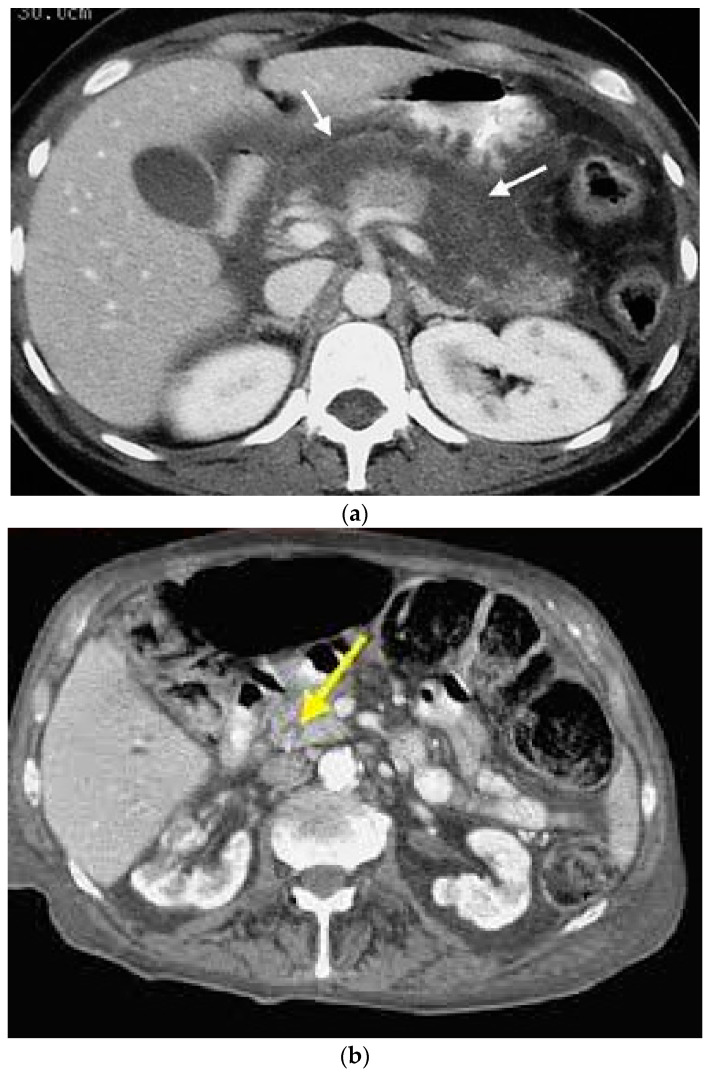
(**a**,**b**) describe acute edematous pancreatitis (arrow) associated with distended gallbladder and the presence of a 7 mm stone fragment in the ampulla of Vater.

Complications of CDL, in turn, include acute pancreatitis (AP) and cholangitis (AC), among others [36]. These conditions increase morbidity and health care expenditures, highlighting the importance of an early and accurate diagnosis. The most common complications of ACC [37,38,39] are the following:

o Mirizzi’s syndrome [40]: rarely, a stone impinges on the cystic duct and compresses and obstructs the common bile duct, causing cholestasis.
o Acute biliary pancreatitis [41]: gallstones pass from the gallbladder into the biliary tree and block the pancreatic duct (Figure 2a,b).
o Cholecystoenteric fistula: rarely, a large stone erodes the wall of the gallbladder, leading to the creation of a fistula with a loop of the small intestine (or elsewhere in the abdominal cavity); the stone may progress freely or obstruct the small intestine leading to a biliary ileus condition.

In contrast, as for ACC without gallstones, the symptoms [42] are similar to those with gallstones, but it could be difficult to identify because patients tend to be critically ill (e.g., in intensive care units) and may not be able to communicate clearly.

Abdominal distension or an unexplained fever may be the only signs.

## 4. Disease Course and Tokyo Guidelines

If left untreated, the disease can rapidly progress to gallbladder gangrene and its perforation, causing sepsis, shock, and peritonitis; mortality approaches 65%.

The progression of ACC can take different forms, from mild inflammation, treatable with oral antibiotics, to severe forms with septic shock or biliary peritonitis, requiring specific treatment [43,44]. However, until 2007, there was no clear criteria for defining ACC and assessing its severity. It seemed necessary then to standardize the treatment strategy for ACC and establish common criteria for subsequent management. Thus, in 2007 an international conference related to gallbladder disease was held in Japan and resulted in a set of recommendations, thenceforth referred to as the “Tokyo Guidelines” [45]. These guidelines (Table 1) were recently updated in 2018 [46] with a review work of 216 articles related to the diagnostic criteria and severity grade of acute cholecystitis, started in 2016. Based on these articles, the newly collected evidence on the diagnostic criteria and severity grading of acute cholecystitis of TG13 was released. It was noted that, indeed, the diagnostic criteria are relatively clear and most studies are instead about severity classification [47,48,49].

## 5. Diagnosis

Most gallstones are asymptomatic. In fact, in most cases, the disease is identified by abdominal imaging, performed for other medical reasons or even found incidentally during a laparotomy [1]. Practically, for the gallstone disease to become symptomatic, it will have to wedge into a visceral structure, such as the cystic duct. In contrast, stones do not give any symptoms when they do not obstruct the duct or biliary tree.

**Figure 3:**
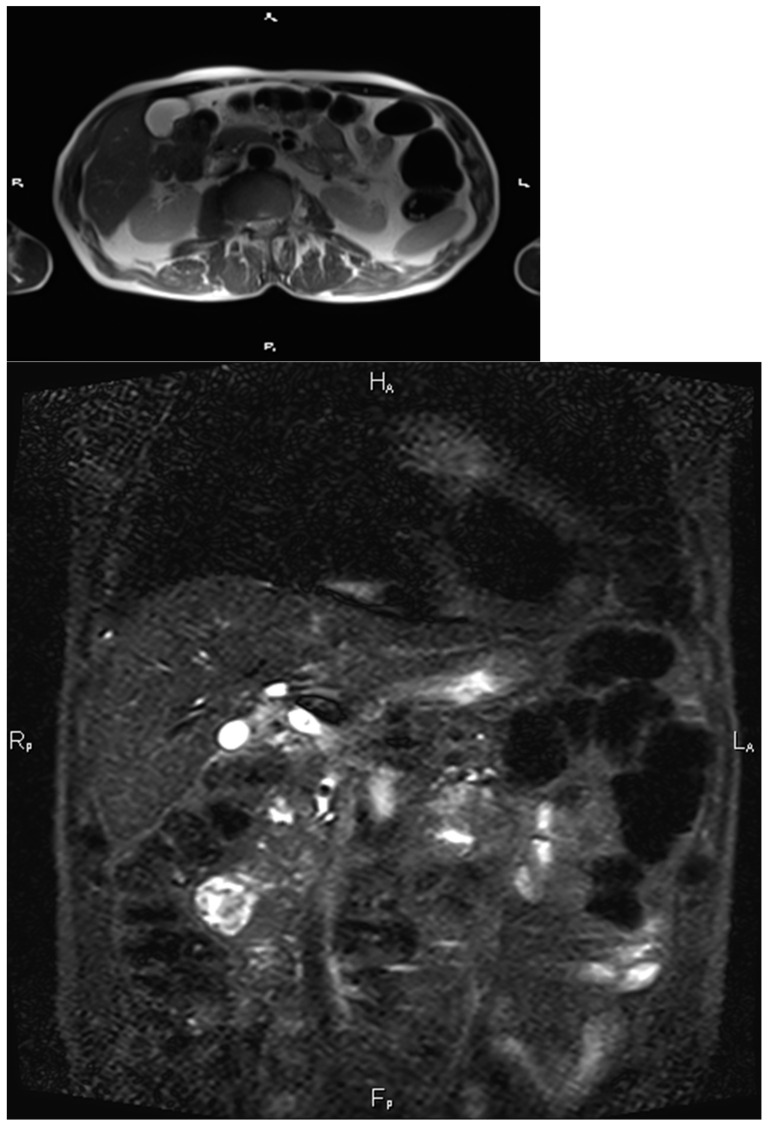
Magnetic resonance imaging of choledocholithiasis. Case courtesy of Mohammadtaghi Niknejad. From https://radiopaedia.org/?lang=us (accessed on 21 February 2023).

CBD stone risk stratification remains controversial, and a more accurate algorithm using clinical features is still needed. The cost-effectiveness of such algorithms must also be evaluated. There is a need to establish a simple, non-invasive, and cheap diagnostic method to identify patients with CBDS for further evaluation with invasive and more expensive techniques. Concomitant CBDs are present in 10–20% of patients with gallstones. The high-risk CBD stone criteria, as per the American Society for Gastrointestinal Endoscopy (ASGE), are one of the following: (1) CBD stones evident on US or cross-sectional imaging; (2) total Bb level > 4 mg/dL and a dilated CBD; or (3) ascending cholangitis [50,51]. High-quality observational studies have shown that in patients with abdominal or gastrointestinal symptoms with a suspected biliary lithiasis it is absolutely necessary to look at liver function tests (LFTs) and abdominal ultrasound (US). In comparison, other observations have established that a cholangio-magnetic resonance imaging (MRI) should be performed (Figure 3) if US does not detect stones in the choledochal, but the biliary tract is dilated and/or LFTs are altered [48]. This applies also if cholangial-Wirsung-MRI does not allow a definite diagnosis to be established.

Some studies reported that the disease severity plays a useful role in predicting vital prognosis [49], as well as the duration of hospitalization and the rate of conversion to laparotomy, the latter being significantly higher in more severe cases [50,51]. In other studies, however, severe cholecystitis may be drained; although, percutaneous cholecystostomy is not always feasible, and open cholecystectomy may be necessary [52,53].

Endo et al. performed a multivariate analysis of “big data” and used the results to propose a new treatment strategy for grade III according to the TG13 severity classification [54]. Although the prognosis of ACC is most generally good, the survival prognosis is still determined by the severity grade.

Recently, endoscopic ultrasound (EUS) is considered as an important diagnostic tool for gallbladder disease [55], although there is no specific indication on EUS in AC diagnosis, with other modalities more cost-effective in this setting. However, a possible application of EUS includes the advantage of having a single diagnostic-therapeutic procedure for some acute cholecystitis cases where a surgical approach would be postponed, for example, because of an unfit patient [56].

## 6. Antibiotic Treatment

Although milder forms of ACC can be treated without specific microbial culture results, it is recommended to undertake direct biliary cultures following percutaneous drainage or surgery, particularly in cases of severe ACC (grade B recommendation) [57]. A study conducted in 175 Dutch hospitals [58] showed that the antibiotic chosen for biliary surgery was ineffective against bacteria in the bile in 23% of cases. Instead, it was determined that blood cultures are of more limited value in ACC because they are rarely positive and, with respect to those guidelines, it is recommended that a separate gallbladder wall fragment be used for culture and histology.

In fact, whether there is an actual relationship between infected bile and post-operative complications is still debated [59]. So far, one study in particular conducted on 213 patients undergoing cholecystectomy for either acute or chronic gallstone disease found no difference in wound infection rates between patients operated on for ACC and those operated on for chronic cholecystitis, which would mean that the presence of bacteria in the bile does not seem to influence the wound infection rate in either group. However, there are two other studies that correlate more severe ACC with positive bile culture [60,61]: patients with cholangitis were more likely to have bacteribilia and anaerobes in the bile. A total of 29 of the 33 patients with cholangitis took broad-spectrum antibiotics for at least 4 days, including an aminoglycoside, before surgery. Practically, it was seen that patients with preoperative cholangitis had no greater propensity to develop infectious sequelae or biliary complications. However, patients with cholangitis were much more likely to develop increased serum creatinine, which, in turn, contributed to longer post-operative hospitalization. Because aminoglycoside therapy may have contributed to post-operative morbidity and a longer hospital stay, aminoglycosides should be reserved only for patients with the most severe cholangitis and should be used with great caution.

To date, the use of antibiotics for gallstones disease is a matter of debate [62]. The treatment of cholecystitis with antibiotics is often mandatory since recently it has been shown that most of the causes leading to the development of cholecystitis are determined by pathogenic bacteria that enter the biliary excretion system with blood or lymph from other organs, either via the downlink or uplink pathway from the digestive tract.

Antibiotics in this circumstance are mandatory, as reported in Table 2. If the source of the infectious process is not eradicated, the disease can be complicated by abscess formation or suppuration of the gallbladder and ducts, which can later lead to a fatal outcome. There instead is no scientific evidence of the validity of antibiotic therapy for asymptomatic ACC.

Regarding preoperative antibiotic therapy, it must be considered when the patients have specific characteristics, as well as age older than 60 years and diabetes [23,63]. Thompson et al. [25] correlated the ACC occurrence with fever > 37.3 °C, total bilirubin > 8.5 mg/dL, and leukocytosis > 14,100/mm^3^ in 63% of patients who had infected bile, which was significantly higher than in 6% of patients with none or only one of these three criteria, as well as a Japanese study about ACC’ guidelines confirmed too [64]. The “Tokyo Guidelines” [53] recommend starting antibiotics after obtaining blood cultures if the temperature is above 38.5 °C and in all elderly or immunocompromised patients, but in clinical practice, blood cultures are rarely performed. However, antibiotic administration before CCY for ACC was shown to reduce the rate of wound infection and post-operative bacteremia [23].

A randomized trial [65] looked at 84 patients, 42 of whom were given preoperative antibiotic therapy with amoxicillin/clavulanic acid, and then underwent delayed CCY, e.g., at 6–8 weeks after the diagnosis of mild ACC. The length of hospitalization and readmission rate were not affected in the 42 patients who received antibiotics, compared to the 42 patients who did not receive any preoperative antibiotics. The duration of hospitalization and readmission rate (primary endpoints) were not changed in the 42 patients who received antibiotic therapy.

Regarding peri-operative antibiotic therapy, because control of the infectious source is essential for the treatment of serious infections, intraoperative antibiotics simply prevent the outbreak of a possible infection by creating a shield around the patient during anesthesia, as with any other type of surgery [65,66]. The PEANUT II trial [19] looked at peri-operative antibiotic prophylaxis and found that the no-prophylaxis group had a higher rate of surgical site infections. In agreement with the results of the PEANUTS II multicenter trial, we recommend antibiotic prophylaxis for complicated acute cholecystectomy requiring surgical intervention. In this case, a sample of bile should always be sent for microbial cultures to identify aerobic and anaerobic bacterial organisms to administer the proper therapy. Currently, there is no scientific evidence of an empirical antibiotic therapy for patients in which acute cholecystitis is suspected, but who have no complications. These patients are encouraged to introduce light meals low in animal fats and avoid spices, alcohol, carbonated drinks, and chocolate, preferring vegetables.

According to Altemeier’s classification, as represented in Table 2 [67,68], which describes microbiological contamination of the surgical site at the time of incision, the presence of infected bile increases the risk of contamination of the cholecystectomy for ACC from clean-contaminated (Class 2) to contaminated (Class 3); therefore, while simple peri-operative antibiotic prophylaxis is done for Class 2, a full course of antibiotic therapy is preferred for Class 3 surgeries. Therefore, given the high risk of infected bile in ACC, antibiotics should be continued even after surgery.

Currently, there is no univocal scientific evidence to recommend the prolonged use of antibiotics. The continuation of antibiotic therapy may unnecessarily prolong the duration of hospitalization, increase the cost, as well as promote the selection of multi-resistant bacteria. A national multicenter study sponsored by the University Hospital of Amiens is currently underway and aims to evaluate the role of post-operative antibiotics in mild to moderately severe ACC [69,70].
Class I/CleanAn uninfected operative wound where there is no inflammation; the respiratory, alimentary, genital, or uninfected urinary tract is not entered. In addition, clean wounds are primarily closed and, if necessary, drained with closed drainage. Operative incisional wounds that follow no penetrating (blunt) trauma should be included in this class, whether it meets the criteria or not.Class II/Clean-ContaminatedAn operative wound in which the respiratory, alimentary, genital, or urinary systems are under controlled conditions and without unusual contamination. Specifically, in this category, biliary tract, vagina, appendix, and oropharynx surgery can be included, provided no evidence of infection or major break in a sterile technique is encountered.Class III/ContaminatedThis is the class that considers wounds contaminated. Fresh, open wounds, which may result from an insult to sterile techniques or leakage from the gastrointestinal tract into the wound, belong to this class. In addition, incisions that result in acute purulent inflammation or without pus are to be considered Class 3 wounds.Class IV/Dirty-InfectedWounds considered absolutely infected. These are typically traumatic wounds that have been treated incorrectly and in an inappropriate manner. They are characterized by devitalized tissue and are most commonly caused by microorganisms present in perforated viscera or in the surgical field.

### Choosing the Right Antibiotic

Choosing the initial antibiotic therapy is fundamental, because it is an independent predictor of mortality [71]. In addition, its prognostic impact is important, particularly in the case of severe sepsis. The main factors to consider are the sepsis site, the severity of the clinical manifestations, the bacteria typically present in the biliary tree, and the nosocomial or community nature of the infection, as well as whether there was antibiotic administration prior to intervention, the characteristics of the individual patient, bacterial colonization, pharmacokinetics, pharmacodynamics, treatment side effects, and antibiotic sensitivity test results [72].

The choice of antibiotic can also be guided by Gram staining of specimens taken in the operating room.

It is recommended to always start by choosing the less expensive, but effective, antibiotic.

In the context of ACC, treatment should start with antibiotics that have good biliary distribution and good activity against the most commonly involved bacteria, as mentioned above [73]. Penicillins are often used in biliary infections: aminopenicillins, such as amoxicillin, are excreted unchanged in the bile. Among the penicillins [74], piperacillin is the one that achieves the best results. In patients with normal biliary function, the concentration of amoxicillin is three times higher in bile than in plasma. Fluoroquinolones exhibit excellent bioavailability with both renal and hepatic excretion. For example, the biliary concentration of ciprofloxacin is 28 to 45 times higher than the plasma concentration. The antibiotic concentration in the bile remains high even in patients with gallbladder obstruction. The combination of ciprofloxacin and metronidazole may be an alternative to the use of amoxicillin/clavulanic acid in patients with mild or moderate ACC and without resistance risk factors [75].

It was seen that tazobactam and piperacillin could also be useful if administered together, but the former has different pharmacokinetics than piperacillin, reaching an effective concentration in the bile only during the first 3 h after administration. Glycylcyclines, on the other hand, have a broad spectrum of activity and good availability in bile [76]. Since most cephalosporins, penicillins, aminoglycosides, and carbohydrates are excreted by the kidneys, their dose should be reduced in patients with impaired renal function. Aminoglycosides are “concentration-dependent” antibiotics; they should be administered in a single daily dose in most cases. While bactericidal activity correlates with peak concentration, toxicity correlates with the residual level or trough level.

To optimize their use, peak serum concentrations and serum concentrations of aminoglycosides should be routinely monitored.

Nine randomized trials and two comparative studies looked at antibiotics such as piperacillin, ampicillin in combination with an aminoglycoside, and third- and fourth-generation cephalosporins. Yet, these data are old and involve antibiotics no longer used in clinical practice. Comparing results is difficult because the populations studied were not composed exclusively of ACC patients, and therefore there is a methodological bias. However, these series demonstrated that a number of different antibiotic regimens were equivalent to the combination of ampicillin with an aminoglycoside antibiotic, considered the standard regimen for ACC in the last decades [77].

It is very likely that some patients with mild, minimally symptomatic symptoms could be treated without antibiotics, if an early (<72 h) CCY is performed. For ACC with more severe symptoms, the proposals for antibiotic therapy are detailed, depending on the severity of the clinical picture and the presence or absence of risk factors for infection with beta-lactamase-resistant organisms. However, the national guidelines of the French Society of Anesthesiology mirror those proposed by the American Society of Infectious Pathology and the World Society of Emergency Surgery [78,79,80].

In general, clinical experience [80] was the basis for the duration of antibiotic therapy for many years, despite the fact that there was no scientific evidence to support it.

## 7. Surgical Treatment

When acute cholecystitis is suspected because of alterations on LFTs without major abdominal or gastrointestinal manifestations, or when gallbladder and biliary tract US of the gallbladder are within the normal range, the patient should be informed that treatment is required in case of symptoms only, but in the meantime, it is recommendable to follow dietary advice for a light meal intake with poor animal fat content. Overuse of carbohydrates also predisposes to cholecystitis: if ingested in large quantities, they lead to significant weight gain, and, as mentioned above, obesity is closely related to the occurrence of gallstones. It should be noted that according to the most recent literature, the natural history of asymptomatic CBDS is controversial, with no definite indication for treatment. It is also of note that ACC begins to improve after 2–3 days and resolves within 1 week in 85% of patients, even in the absence of treatment; yet, very often, a delay of 24–36 h is necessary to let the patients become fit for surgery. In the early 2000s, Collins et al. reported that stones in one-third of CBDS patients would pass spontaneously within 6 weeks after cholecystectomy (CCY) [1]. Neither stone size nor risk factors for complications of untreated CBDS have been determined. However, complications of ductal stones include a variety of clinical conditions that can be life-threatening, which is why treatment of CBDS is always recommended, except in high-risk patients, in whom a conservative approach is preferable [14]. There is also a category of patients for whom prophylactic cholecystectomy should be considered because of the comorbidities, such as sickle cell and hemolytic anemia, that carry out a much higher risk of symptoms deterioration and contribute to pigmented stone formation, more often resulting in acute cholecystitis.

Before the advent of minimally invasive surgery, open CCY with choledochotomy and/or surgical sphincterotomy plus bile duct clearance was the gold standard treatment of concomitant cholelithiasis and choledocholithiasis [81]. Over the past 30 years, with the introduction of laparoscopic standardized techniques [82] and improved endoscopic diagnostic and therapeutic procedures, new approaches to combined pathology have emerged [83]. Current treatment approaches, which vary depending on a center’s experience and availability, include open or laparoscopic CBD exploration, combination laparoscopic cholecystectomy (LC) with endoscopic retrograde cholangiopancreatography (ERCP), and laparo-endoscopic rendezvous (LERV) [84,85]. Therefore, while LC is commonly considered the treatment of choice for gallstones limited to the gallbladder, there is no consensus on the optimal management of combined cholecysto-choledolithiasis.

If cholecystitis is accompanied by the formation of stones in the gallbladder and/or ducts, the risk of damage and inflammation of the organ wall increases several times, as the concretions can mechanically damage the tissues.

Even in high-risk patients with ACC, laparoscopic CCY is superior to percutaneous catheter drainage. According to a multicenter randomized clinical trial [86], CCY not only reduces the rate of major complications, but also diminishes the utilization of healthcare resources and costs by more than 30%. Our approach is to reserve percutaneous drainage, only in the impossibility of performing surgery immediately, as in fact, all these patients are especially at risk for recurrent gallstone-related disease and should undergo CCY as definitive treatment [87].

Finally, regarding the use of antibiotics after CCY, there is no consensus about the length of time for the correct administration of antibiotic therapy for ACC. To date, only one prospective study [80] conducted on patients with ACC compared two post-operative antibiotic regimens for patients undergoing cholecystectomy. Practically, 2 g of cefamandole were administered to all patients, who numbered 203, before surgery. Instead, after surgery, the patients were divided into two groups: a short course of antibiotics with 500 mg intravenously at the 6th and 12th hour, versus a long course, using cephalosporin for 7 days. The main result was that the duration of hospitalization was significantly longer in patients taking cephalosporin for one week, with, moreover, no significant benefit in terms of surgical site infection.

Recently, in clinical practice, there has been a shift to reduce antibiotic treatment more and more in almost every branch of medicine, and even more in surgery. This has also occurred in the case of gallbladder disease, precisely to try to reduce the selection pressure on antibiotics, fueling the development of the emergence of resistant bacteria. The surgical treatment of ACC has undergone a marked change in recent decades. The use of real-time US and other diagnostic imaging for hepatobiliary excretion has made the diagnosis of ACC very accurate, thanks to its high sensitivity and specificity. Many clinical studies have shown that the morbidity and mortality rates of early versus delayed CCY for ACC are similar, but early cholecystectomy has the advantage of a shorter hospital stay, so it is more cost-effective. In addition, early CCY solves the problem of recurrent colic attacks straightaway, considering also that the organ would be damaged if not promptly treated and, in most cases, as mentioned above, also infected. This eventually also increases the risks of surgical complications [8,88,89,90].

What is being sought, however, is to figure out what is the most appropriate time at which to intervene surgically in early cholecystectomy [91]. Surgical intervention on cholecystitis detected via laboratory test, but in the absence of symptoms, before secondary bacterial infection, will undoubtedly reduce the possibility of post-operative septic complications [92,93].

The diagnosis is often delayed either because the patient does not accept the doctor’s advice or the surgeon is not consulted early enough. It is important that the choice of surgery is considered with reference to the time of onset of the attack rather than the time of admission to a surgical ward. Recommendations on the timing of early CCY surgery range from within 2 days, within 3 days, and within 7 days of symptom onset. It was seen in the study conducted by Lau et al. that the peri-operative administration of short courses of antibiotics makes early cholecystectomy for acute cholecystitis even more cost-effective [83,94,95,96], and the efficacy has been found to be the same as when long courses of conventional antibiotics are given to reduce post-operative septic complications. It also contributes to cost reduction, a reduction in the risk of adverse effects of antibiotics, lowered incidence of thrombophlebitis secondary to intravenous antibiotic administration, and a shortened hospital stay [97].

## 8. Conclusions

All patients with ACC before surgical treatment should be treated with an antibiotic that targets enteric cells and the cells that compose the gallbladder wall, except in patients who have minimally symptomatic benign ACC. In addition, a bile culture sample should undergo laboratory examination, specifically for the selection of the most appropriate antibiotic. The experts also agree that a randomized trial is needed to find the appropriate pre-operative and post-operative antibiotic therapy management guidelines, considering that the latter currently does not seem to be necessary after early CCY, except in very severe cases of ACC, as confirmed by the management in clinical practice and experience of the team in charge.

## Figures and Tables

**Table 1:** Tokyo Guidelines indicating the severity of acute cholecystitis.

Grade	Description
Mild	Patient with acute cholecystitis with no organ dysfunction and mild gallbladder inflammation.
Moderate	The presence of one or more factors among: Increased white blood cells (>18.000 cells/mm^3^); Palpable mass in the right upper quadrant of the abdomen (between hypochondrium and flank); Pain duration > 72 h; Signs of local inflammation, i.e., pericholecystic abscess, hepatic abscess, biliary peritonitis, gangrenous cholecystitis, emphysematous cholecystitis.
Severe	The presence of one or more factors among: Neurological disorders; Cardiovascular disorders (hypotension requiring treatment with dopamine 5 μg/kg per minute or any dosing of dobutamine).

**Table 2:** In this table are summarized the clinical features and symptoms for which it is recommended to immediately begin antibiotic therapy for patients with acute cholecystitis.

Immediate Indications for Antibiotic Therapy for Acute Cholecystitis
Pronounced pain in the liver, with a tendency to increase
A significant increase of temperature (up to >38.5–39 °C)
Expressed digestive disorders, with recurrent diarrhea and vomiting
Pain spreading to the whole abdomen (so-called “poured” pain)
Presence of other infectious diseases in the patient
Infective process signs, detected as a result of a blood test

## Data Availability

No new data were created or analyzed in this study. Data sharing is not applicable to this article.

## Abbreviations

AC	Acute cholecystitis
AP	Acute pancreatitis
ACC	Acute cholecystitis
CBD	Common bile duct
CBDS	Common bile duct stones
CCY	Cholecystectomy
CCK	Cholecystokinin
CDL	Choledocholithiasis
ERCP	Endoscopic retrograde cholangiopancreatography
EUS	Endoscopic ultrasound
LC	Laparoscopic cholecystectomy
LERV	Laparo-endoscopic rendezvous
LFT	Liver function test
MRI	Magnetic resonance imaging
US	Ultrasound
